# Views and practices on medical cannabis of unlicensed providers in Thailand: a qualitative study

**DOI:** 10.12688/f1000research.110367.1

**Published:** 2022-03-29

**Authors:** Sawitri Assanangkornchai, Darika Saingam, Kanittha Thaikla, Muhammadfahmee Talek

**Affiliations:** 1 Department of Epidemiology, Faculty of Medicine, Prince of Songkla University, Hat Yai, Songkhla, 90110, Thailand; 2 Research Institute for Health Sciences, Chiang Mai University, Chiang Mai, 50200, Thailand; 3 Faculty of Nursing, Prince of Songkla University, Pattani campus, Pattani, 94000, Thailand

**Keywords:** Medical cannabis, illegal providers, prescription practice, in-depth interview, legalization, decriminalization, thematic analysis, folk healers

## Abstract

**Background:** Despite the legalization of cannabis use for medical purposes in Thailand in February 2019, illicit providers are still widespread and accessible. This study aimed to understand why people still chose to receive medical cannabis treatment or products from unlicensed or illegal providers. The practices of unlicensed or illegal providers in provision of medical cannabis products or treatment services were also examined.

**Methods:** Qualitative in-depth interviews were conducted among medical cannabis providers and users, including 36 unlicensed and 7 licensed providers and 25 users in 2019-2021. Snowball sampling was used to recruit participants until saturation of data was achieved. Interviews included open-ended questions about the providers’ practices and attitudes towards medical cannabis. Interviews were recorded and transcribed, and thematic analysis was performed.

**Results:** Overall, six reasons were identified to answer why unlicensed/illicit providers were still popular, including: 1) easy accessibility; 2) familiarity with the unlicensed providers before the legal scheme became available; 3) favorable characters (kind, supportive, non-judgmental) of unlicensed providers; 4) affordable treatment fees; 5) trust in the quality of the medicines; and 6) lack of knowledge and negative attitudes towards cannabis from healthcare professionals. Most providers started their career as medical cannabis providers by using it themselves or with their relatives and being satisfied with the results. They used cannabis products to treat all diseases, including skin, eyes, HIV/AIDS, non-communicable diseases and all kinds of cancers. Additionally, they believed that it was effective, with no or minimal adverse effects.

**Conclusions:** This study suggests that some patients will continue receiving medical cannabis treatment and products from unlicensed or illegal providers. More attention should be paid on increasing the capacity of medical cannabis service systems within public health hospitals, and the certification of unlicensed providers, so as to integrate them into a regulated system.

## Introduction

Legality of cannabis use in Thailand has undergone notable changes in recent years. Medical use of cannabis was legalized in February 2019. Nonetheless, cannabis remained an illegal drug until it was recently delisted in the newly amended Narcotics Code which went into effect on December 10 ^th^, 2021. On January 25 ^th^, 2022, the Narcotics Control Board approved the removal of parts of cannabis plant with no more than 0.2% tetrahydrocannabinol (THC) by weight from the Food and Drug Administration’s list of controlled drugs. The decision was approved by Parliament, and the Public Health Minister then signed the announcement of the delisting, which would take effect 120 days after the announcement was published in the government gazette.

After the legalization of cannabis use for medical purpose, medical cannabis clinics, based on modern medicine and Thai traditional medicine, were piloted in government hospitals in August to September 2019, and scaled-up nationwide in 2020. Licensed healthcare practitioners, including medical doctors, dentists, Thai traditional medicine doctors, and folk doctors can prescribe medical cannabis products, which are registered under the Special Access Scheme (SAS). Three groups of medical cannabis products have been approved for medical use: 1) registered drugs per the new Narcotics Act; 2) Thai traditional medicine with approved compositions (16 regimens), and 3) folk-doctor cannabis oil. Despite this scale up of medical cannabis clinics, access to registered products for patients had been difficult as indications for prescription were limited and regulations regarding possession as well as production of medical cannabis were constricted. However, illicit cannabis trade from illegal medical cannabis suppliers, recreational dealers, and online suppliers, also became widespread and more accessible during this period. ^1^

Previous studies have shown that people use medical cannabis for a variety of health conditions, such as pain, mental health and sleep problems and through its use felt relief of their condition. ^2^^–^^4^ A study in Australia, in the early phase after legalization, indicated that the main sources of supply of medical cannabis were from recreational dealers, friends or family, illicit medicinal cannabis and online suppliers, and by growing their own. ^2^ Furthermore, studies in Canada ^5^ and the United States of America (USA), ^6^ where medical cannabis is legally available, indicated that physicians felt reluctance or ambivalent to authorize cannabis use for their patients, because of either a lack of knowledge or unfamiliarity with pharmacology, formulations, dosing of cannabis, lack of product standardization, lack of research examining the effectiveness and risks of cannabis use, and uncertainty regarding the policies.

To date, no study has examined the views and practices of illicit providers of medical cannabis in Thailand. With an increasing demand of medical cannabis products amidst the restricted access to legal supply in Thailand, unlicensed medical cannabis providers and illegal suppliers come into play as an available source of medical cannabis products. Our study, conducted during the first year after the legalization of medical cannabis use in Thailand, found that 74% of the medical cannabis products used came from illegal sources, such as underground traders, not-for-profit provider groups (for example: priests, folk healers and civil society advocacy groups), friends and relatives, and home or clandestine growers and producers. ^7^ In this study we aimed to understand why people still chose to receive treatment or medical cannabis products from unlicensed or illegal providers, despite licensed medical cannabis clinics in hospitals under the Ministry of Public Health (MoPH) having been opened nationwide since late 2019. We also examined the practices of unlicensed or illegal providers in provision of medical cannabis products or treatment services. In addition, this study examined the perspectives of medical cannabis users with regards to their access, perceived benefits and risks, and satisfaction towards those providers. Information obtained from this study could benefit the medical cannabis health care system in Thailand and other countries that have planned to, or have already initiated, medical cannabis policies. It will help in planning strategies to improve the capacity of said providers, and their services as well as improve access to medical cannabis.

## Methods

### Study design

This study is a part of a larger two-phase study; using a mixed-method approach among medical cannabis users and providers in Thailand. The phase-1 study was conducted between October 2019 and February 2020; the first year of the medical cannabis legalization, followed by phase-2 between November 2020 and February 2021. Both phases comprised of a quantitative cross-sectional study, using respondent-driven sampling among medical cannabis users, and a descriptive qualitative approach involving in-depth interview of medical cannabis providers and users and observation of the providers’ practices and their medicines. Data of the qualitative part of both phases were used for thematic analysis. The descriptive qualitative approach, often used to discover the nature of the specific events under study, allows for a comprehensive summarization of views and practices of illegal providers and their services experienced by medical cannabis users, a topic about which little is currently known. ^8^

### Participants

We included 36 medical cannabis providers, who had not been certified by the MoPH as licensed folk doctors in this study. They included 15 folk healers, 9 growers or clandestine producers, who also provided treatment and counseling on using their products, and 12 workers of civil society networks or social media administrators that provided medical cannabis products and advice. Folk healers wishing to get prescription licenses need to be certified by the head of the provincial public health office or the Department of Thai Traditional Medicine and Alternative Medicine of the Ministry of Public Health. Those that meet the following criteria can be nominated by the village committee or local administrative organization for certification: aged at least 35 years, living in the community where the nomination takes place for more than 10 years, having knowledge and competence in promoting and caring for the health of people in the community using Thai traditional medicine wisdom according to their community culture for more than 10 years with admiration of the people in that community, being sane of mind and never having been incarcerated. Although, some of our participants had been practicing as folk healers for many years, they had not been certified; due to inadequate eligibility criteria or they just did not want to; said participants were recruited as unlicensed providers. Participants were eligible for the study if they were aged 18 years or over and willing to participate in the study. Exclusion criteria were set as being intoxicated, cognitively or mentally impaired, or too ill to be interviewed. However, we did not exclude any subject because of any of these reasons.

Purposive sampling was used to recruit participants. First, some key informants, e.g., folk healers who practiced in the community and workers in non-government cannabis advocacy organizations were identified. These informants were then asked to provide contact information of other providers, which could be approached for an interview. In addition, some medical cannabis users, participating in this research, also provided us with the contact information of their providers.

In addition, seven licensed providers, including five medical doctors and two Thai traditional and alternative medicine doctors were also interviewed. Further, 25 medical cannabis users were recruited through snowball sampling, starting from some well networked individuals who were known by the researchers as being medical cannabis users. Participants were recruited until enough participants had been interviewed to achieve saturation of data. ^9^

### Data collection

Participants were first contacted by telephone and invited to participate in the study. Each of four research assistants, who were at least bachelor’s degree graduates with previous experience in qualitative data collection with people who use drugs or people living with HIV in our other research projects, together with DS, KT or MT, who were experienced qualitative researchers, then visited the participants at a location set by them, e.g., home or workplace, and conducted the interviews.

Before the interview, verbal informed consent was obtained, and all interviews were audio recorded. At the times of data collection, most providers and consumers of medical cannabis were considered illegal, the use of written consent form might be perceived by participants to be threatening and treated with considerable skepticism by some participants. Signed informed consent form is the only record linking the subject and the research, and the principal risk would be potential harm resulting from a breach in confidentiality. To ensure the anonymity of participants, eliminating the risk that signatures could be linked to responses, verbal informed consent was obtained before the interview. In addition, the interview involved no more than minimal risk to subjects; therefore, the waiver of document of consent did not affect the rights and welfare of the subjects. Our Institutional Review Board thus waived the requirement for documentation of informed consent and allowed for verbal informed consent for both phases of the study.

The interviewers also made notes on nonverbal communications, which were used to supplement the audio-recorded information during transcriptions to ensure extensiveness of data. The interview was conducted in private and took up to one hour. Some follow-up interviews were also conducted for participants whose data from the initial interview were not complete, or when more clarification was required. The interview guide with open-ended questions and themes developed by the research team was used. We first tested questions included in the interview guide with some medical cannabis providers, such as two folk healers and two staff of not-for-profit medical cannabis organizations, and two users of medical cannabis. The guide covers content on the participants’ practicing experiences in providing, producing or using medical cannabis, source of cannabis, knowledge regarding diseases treated with cannabis, how they obtained this knowledge, and opinions towards medical cannabis laws and policies in Thailand. The guides can be found as *Extended data.* ^19^ DS, KT and MT participated in the interviews and supervised the process of data collection and data transcriptions.

### Data analysis

All interviews were transcribed verbatim by the research assistant who did the interview. DS, KT and MT also listened to some randomly selected interview recordings while reading through the respective transcribed data and field notes to ensure completeness and accuracy of the transcriptions. Qualitative data analysis was conducted manually. DS and SA then read the interview transcripts and notes repetitively, coded and aggregated transcribed text into meaningful themes and subthemes. The other members of the research team then read and discussed initial themes and subthemes until agreement was reached. For each subtheme, supporting quotes were selected to illustrate key points in the findings.

### Ethical approval

Both study phases were approved by the Research Ethics Committee (REC) of Faculty of Medicine, Prince of Songkla University [REC.62-205-18-1, dated 7 October 2019] and [REC.63-449-18-1, dated 3 December 2020]. The approval of the waiver of written consent was also documented in the REC approval documents.

## Results

Altogether, 36 unlicensed or illegal providers, 7 licensed providers and 25 users participated in the study. Some participants initially refused or were reluctant to be interviewed; however, after having been given a detailed explanation of the study, including objectives and confidentiality safeguards by key informants in their community, all agreed to participate. The unlicensed provider sample included 34 men and 2 women, who had been involved in medical cannabis provision for a median of 50 years (range 25-85 years). Three of them were Buddhist monks. The users were 14 men and 11 women, whose age ranged between 32 and 80 years, and had been using medical cannabis for treatment for a variety of conditions, such as cancer, hypertension, migraines, insomnia and stress for a period of 2-10 years.

### Why unlicensed/illicit providers were still popular

Overall, six main reasons were identified for people choosing unlicensed providers and products: 1) easy accessibility to unlicensed or illegal sources; 2) familiarity with the unlicensed providers; 3) favorable characters of the providers; 4) affordable treatment fees; 5) trust in the quality of the medicines; and 6) a lack of knowledge, confidence and negative attitudes towards cannabis from healthcare professionals.

*Easy accessibility.* Although medical cannabis clinics have been opened nationwide indications for treatment with medical cannabis oil extracts are limited, and accessibility has been poor and slow. Therefore, unlicensed or underground providers, who were more easily accessible, became their best available choice. Folk healers usually opened their practice within their own home where patients could visit them anytime without prior appointment. Some providers allowed their patients to contact them by Line application or telephone for consultation concerning health problems and medication adjustment; making patients feel supported and confident. Some even provided home visits or home delivery of medicine to their patients with limited mobility, such as the elderly or those with physical disabilities.

*Some patients cannot come by themselves, so they ask their children or caretakers to fetch medicines for them. They can come any day, or at their convenience. Some patients could not come up to my cubicle, so I went down to see them in their cars. When patients or their relatives come, we never refuse to see them or tell them to go home; even when their conditions are beyond treatment. (FD01)*

*Some hospitals limited the number of patients to as little as 5 per day, and they are not open every day; maybe even only one day per week. (FD02)*

*After five weeks of the clinic opening, we have seen 127 patients; however, only 48 cases have received cannabis medicine, because the others did not fulfil the indications. Many came because of insomnia, which does not fit the indication. Most have cancers; for example, lung, stomach and colon, with metastasis to other organs, but they are still in stage 3 which is not an indication; so, we cannot give them cannabis. The others have Parkinson and Alzheimer, for which they cannot receive cannabis oil either, because we have only THC oil. (MD01)*

*I know I can get cannabis oil from the hospital, but I don’t want to go to the hospital. Going to a hospital is complicated. (PT01)*

*Familiarity.* In some areas, folk healers had been well-known and accepted long before the boom of medical cannabis use in modern society. Using cannabis plants in folk and traditional medicine regimens has been regarded as ancient Thai wisdom. These folk healers, therefore, had already had follow-up with their patients for many years, and these patients preferred to continue treatment with their respectful and trustful doctors, rather than changing to new doctors in MoPH hospitals.

*Most of my patients are local people living in this village, so we meet when we make merit at the temple regularly. I visit my patients at home every week. I do it as a routine. For some families I take care of the whole family. (FD03)*

*I am confident and trusting in … (a popular provider in the area)’s cannabis oil. I have been using it for more than one year. I have had migraines for 20-30 years. I had used medicines obtained from the hospital for several years, but they did not work. Two weeks after I took cannabis oil, I felt better so I continue using it. Why should I waste my time and pay bus and ferry fees to go to the hospital, while I can just ride a motorcycle or call …’s team to deliver the oil for me? (PT03)*

*Providers’ characters.* The folk healers that were interviewed appeared to be natural counselors, who understood and empathized with their clients’ illness and suffering. They were volunteer-minded and had the same goal as that of to help people. Patients also found the folk healers to be non-judgmental and non-stigmatizing. Almost all folk healers did not record their patients’ information systematically and did not make follow-up appointments with their patients. They just memorized the information and told patients to come back as needed, or they visited their patients at home when convenient. This surprisingly made patients more comfortable, as their information was confidential.

*Why patients are getting well is not only because of the medicines, but it is also the conversations between patient and provider. It is a positive energy. They can talk with us through the chat box. We cheer them up and encourage them to fight the disease (NGO01).*

*I never record patients’ information, and never give patients’ information to anyone. They trust me and can call me anytime. Some relatives call me late at night; telling me that the patient cannot tolerate anymore. I encourage them and tell them to come in to take cannabis oil. (FD03)*

*He (a respectful monk) is very kind. He always asks about my symptoms and if I have any side effects of chemotherapy after using cannabis oil. He advises me about diet, selfcare and teaches me some dharma (Buddhist teachings) too. (PT04)*

*He (a cannabis oil producer) visits me regularly, brings me the cannabis oil and some snacks. He knows that I live by myself, so he comes very often. When I got sick, he is the one who took care of me. I feel happy, laughing and not stressed when he comes. (PT05)*

*Folk doctors never refuse us. They are always ready to give help and good advice for us to fight. (PT06)*

*Affordability.* Some folk healers - for example, Buddhist monks and those working in some civil society not-for-profit organizations - provided medical cannabis products free of charge for those who could not afford to pay. Folk healers who have been practicing in a conventional way do not usually ask for treatment fees; they accept only a “teacher worship fee”, which is very small. Although medical cannabis treatment in the MoPH hospitals was also free, as it was covered under the universal coverage or other medical insurance schemes, patients had to pay the transportation fee by themselves. Additionally, at the time of data collection of this work, MoPH medical cannabis clinics had yet been opened in every province, so some patients had to travel far to receive treatment. Nonetheless, some unlicensed providers charged for their products and treatment cost was very high; especially those who advertised their services and products through social media.

*Our center provides free cannabis oil to both Thai and foreign patients, regardless of their sex, age and socioeconomic status. We send free cannabis oil to every patient’s home and follow them up. … We make a sticker to put on the products that they are free, not for sale and that it is from a not-for-profit organization. Some foreign patients who received treatment from us and were impressed with it donated some money to our foundation, or sent product containers to us depending on their convenience. (NGO02)*

*I teach patients and their relatives to make their own cannabis medicine. I told them to secretly grow 2-3 plants and produce their own medicine. I just give advice and follow their symptoms. I cannot take money from them because I don’t buy cannabis. (FD03)*

*I don’t buy cannabis as my friends who grows it gives it to me. Folk doctors exchange their products; for example, I gave my colleagues some herbs that they don’t have to trade with cannabis. We don’t put money value on our herbs. (FD04)*

*Quality of the medicines.* Some patients believed that the medical cannabis oil extract provided from the MoPH hospital was too low in concentration (say, 1.7%) of active ingredients to be effective, while the oil extract from unlicensed sources was of better quality and could treat more diseases. Most conventional folk healers used the parts of raw plants to make their medicine mixture or extracted the crude oil in their home-kitchen. They may also grow, or suggest patients grow their own cannabis plants, to assure quality, and to keep the plants free from contamination. However, some patients as well as providers also worried about the quality of the illegal products as their sources were unknown, so they might be contaminated, and the production process might neither be so qualified.

*I tested cannabis medicine from the Government Pharmaceutical Organization. I think it’s not good. I think I make better products than those of the government hospitals, because I extract it by myself to treat my patients. I tailor make the medicine to the severity of the patients’ conditions. (FD03)*

*Now underground products are of premium grade. Their production technique has gone so far, there are many talented chemists who have ever lived overseas. They want to make it known that the best formula is not what produced by the governmental people. We (underground producers) import extraction machines from China and Switzerland and secretly sent the extracts to some university professors to qualify them. (NGO03)*

*How can we deal with the underground dealers? Some sell fake oil which has no medicinal content at all. (FD03)*

*I am confident in … … (a popular provider in the area), because he extracts it in an organic way. (PT03)*

*Healthcare providers’ lack of knowledge and negative attitudes.* One of the main reasons for medical cannabis being limited in prescriptions at public hospitals was due to the clinicians’ attitudes coupled with their readiness to provide it. Although medical cannabis training courses have been organized for doctors, pharmacists and other healthcare professionals to provide knowledge and grant certification for prescribing medical cannabis since the legalization, not many practitioners attended the courses; hence, most clinicians were not well enough prepared for medical cannabis practice. The general attitudes of medical professionals; in particular psychiatrists and pharmacists, were negative, due to concerns over adverse effects of mental health from cannabis use. Additionally, they were of the opinion that safer and more evidence-based medicines were already available for any indications wherein cannabis was to be used. Most medical professionals learn to practice medicine based on scientific evidence and from what they learn in medical schools. However, medical cannabis was new for them and supporting evidence was still limited, while conflicting evidence of benefits and harms was abundant. They were thus reluctant to prescribe medical cannabis. Moreover, there were strict regulations to follow and many forms to fill out when prescribing cannabis; medical cannabis prescription in a public hospital was still very restricted. An experienced and licensed doctor, who supported medical cannabis, expressed that medical professional might be the one who referred patients to the unlicensed or illegal system, because they refused to learn and prepare themselves to prescribe medical cannabis; despite their full awareness that their patients were using it.

*In our hospital, pharmacists and psychiatrists don’t agree with medical cannabis use. … I think it’s not evidence that makes the resistance, but it’s the mindset. (MD01)*

*We, medical doctors have no right to refuse medical cannabis. We know that our patients use it. If we don’t learn and become aware of it, it means we don’t care for the patients and let them use it without our advice. Now we don’t even know if cannabis is good or bad, but if we refuse to learn and prescribe medical cannabis, it means we push our patients towards the underground system. (MD02)*

*Previously we have learned how to treat other diseases from what we learned in the university. However, for this issue (medical cannabis prescription) we have to learn it by ourselves, and start using it on our own; based on very limited evidence and a two-day training course. (MD03)*

### How unlicensed providers practiced medical cannabis treatment

Six subthemes were derived, including: 1) how they started their career as medical cannabis providers; 2) roles of the providers; 3) health conditions for which medical cannabis was used; 4) types of products and dosing; 5) use of modern medicine while using cannabis; and 6) progression of illness after treatment with cannabis.

*Starting the career.* Most providers started their role as medical cannabis service providers from using cannabis as a self-medication for their or their relative’s health problems. After success in treating themselves or their relatives, they felt confident in using cannabis for other people. Some providers were full of interest and enthusiasm in acquiring knowledge on medical cannabis obtained from international published literature, social media, training courses and actual case studies. Moreover, cannabis has been a medicinal herb in Thai traditional medicine pharmacopeia since ancient times. Folk healers have acquired knowledge regarding medical cannabis from their ancestors, who were often folk healers as well. Therefore, folk healers were knowledgeable and experienced in medical cannabis treatment long before the start of medical cannabis within modern healthcare systems.

*The origin of my work as a medical cannabis provider was because my mother got sick and could not walk. So, I trained from Mr. … (a famous unlicensed provider) and also took a Cannabinoid Medicine Training Course. I started using it with my mom and was very satisfied with the results; my mother can walk again. People are confident in me and ask me to provide treatment for them. (NGO04)*

*At that time there was a social trend that cannabis is a cancer medicine, I sought some information and photocopied the documents to give to my patients and relatives. Firstly, my close relative was sick and had to take a handful of medicines each day. I thought that in not so long his liver and kidney would be damaged, so I told him to try cannabis oil. He got better, blood pressure and sugar decreased. So, after that I advised other people in the area to find good quality cannabis products to treat their diseases. (FD03)*

*I have to try it with myself before using it with my patients, because all cannabis plants are different. (FD06)*

*Cannabis oil is new in modern medicine, but traditional medicine has used it for a long time. All folk doctors know that cannabis is a kind of medicine and use it as an ingredient in several types of medicine; for example: “Happy sleep” regimen helping in sleep and appetite, “Santhakart” relieving constipation. I have cooked these regimens for a long time. (FD07)*

*Providers’ roles.* The providers had varied roles, including providing assessment, treatment and counseling as folk doctors, providing knowledge concerning medical cannabis summarized from published literature, being active advocators for legalizing cannabis, and growing, producing and selling medical cannabis products. Buddhist monks played active roles in not only being a spiritual center for local people, but also providing holistic care to patients, from the beginning to the terminal phase of illness; especially those classified as beyond available conventional treatment. Some providers believed that anyone could be a medical cannabis provider, if they cared about people and continued acquiring knowledge about diseases, by learning from research documents and by observing patients’ symptoms and progression.

*My role is not only a monk who provides spiritual guidance, but also a doctor, pharmacist and counsellor. Patients can telephone me anytime. I advise them to follow religious principles to pray and be mindful on breathing, not to be too worried about the illnesses; as birth, aging, illness and death are a common truth. We encourage them to fight and find something to do. We should think that we are better than many people and well taken care of by our children. (FD05)*

*After seeing a lot of patients, we would know why they do not respond to treatment, know if they use it in a correct way and have the discipline in taking care of themselves. We do not have to be a folk doctor or know everything like a medical doctor. We just know what should or should not be eaten, and that all diseases have different symptoms and stages; then adjust the dosages to best suit the patient’s current condition. (NGO05)*

*Health conditions.* The health conditions for which cannabis was prescribed by folk healers included: cancers of various organs, Parkinson’s disease, epilepsy, muscle aches, headaches, insomnia, stress, depression, nausea, vomiting, psoriasis, acnes, ringworm, hemorrhoid, diabetes, hypertension, gout, HIV, and as a substitute for other drugs of abuse (methamphetamine and crystal methamphetamine). Terminal stage cancers, such as breast and brain cancers were the most common diseases patients sought out for cannabis treatment; especially when they were beyond available modern treatment or when they were to receive chemotherapy or radiotherapy. Their perception was that cannabis would help prepare the body to tolerate the side effects of such modern treatments. Most providers and their clients believed that cannabis could treat all diseases. Some providers indicated that cannabis balanced the system inside human body and could help relieve all symptoms that patients were suffering, for example: pain, fatigue, low appetite and sleep difficulty. If patients improved from these symptoms they would feel well and have the energy to fight the disease.

*Cannabis can treat all diseases; it is the God of all medical herbs. (FD07)*

*Cannabis can treat almost all diseases, say more than 80 diseases. It can also be mixed with many herbal medicine regimens to help patients to get rest and repair their body. Cannabis is a repairer to help us sleep soundly. (FD04)*

*Cannabis oil will control cancer cells, so as they do not proliferate. (FD03)*

*Cannabis has several benefits, especially effects on the nervous system, helping with sleep, dementia and Parkinson, etc. (FD04)*

*I think it (cannabis) helps balance the body. It does not treat a disease but gives immunity to us. Whatever disease we have, if our body is good, it will treat itself. Cannabis helps release a happiness agent, this agent then kills all diseases or suffering agents. Any medicine which makes us happy will balance our body system to fight a disease. (FD09)*

*Products and dosing.* The products forms were various, such as extract oil in liquid form for sublingual administration or in a capsule for swallowing or for rectal suppository, tea made from dried raw plants which was claimed to be a good remedy for insomnia, topical skin cream and soap for skin diseases, toothpaste for toothache and caries, and a mixture bolus of the cannabis plant with other Thai herbs. Some folk healers also prescribed dried plants for smoking. Information related to product forms, route of administration, actions, dosing and sources is widely available on the Internet, through social media and word of mouth, for both providers and patients to learn and adaption of use for themselves, or when prescribing to others.

Some healers advised their clients to start off with a test dose of one small drop of extract oil. If there was no sign of an allergic reaction, the users were advised to step up the dosage slowly until they found a suitable dose for themselves. They were then advised to maintain that dose until their symptoms subsided, then decrease the dose and finally stop when the symptoms disappeared. Females were advised to take a smaller dose than males. Morning and/or bedtime doses were usually recommended. Most providers emphasized that their clients should not take a second dose of oral extract oil within four hours after their first dose. They said that the action of the oral form was slow: approximately 30 minutes; therefore, if the second dose was taken shortly after the first dose their clients could easily get intoxicated. However, in a smoked form it was fast acting; approximately 5-15 minutes; thus, it was recommended for cases of cancer or severe stress, and for those who had pains or sleep difficulties.

*Each body is different. They should try it by themselves, so as to find out how much is suitable for them by measuring from their sleep. If it is too small, we cannot sleep then we can increase the dose; if it’s too much, intoxication will occur, so we should decrease the dose. (FD08)*

*Capsules work with the enzyme system and is good for patients with colon cancer and cancers of the organs of the lower part of the body, such as prostate and ovarian cancers. For brain cancer, I recommend the smoking form with oil extract as cannabinoid glands are in this area. (NGO02)*

*Vaginal cancer patients should use a suppository form before having chemo or radiotherapy. Rectal suppository is good as it is not intoxicating and will revive our liver.*

*Use of modern medicine.* Most folk healers advised their patients to stop or reduce their dose of modern medicine which they had used before. They explained that modern medicine contained a lot of chemicals, causing imbalanced body function, and might impair liver and renal functions. However, some said cannabis and modern medicine should be used together, as cannabis would enhance the effects of modern medicine. Some folk healers viewed that terminally ill cancer patients who required morphine to relieve pain should receive supplementary cannabis, while tapering off morphine until stopping and then maintaining treatment with cannabis alone. Some folk doctors advised their patients to take cannabis and modern medicine at different times, so they would not interact with each other. Some even knew that cannabis was contraindicated for patients with cardiac arrythmia, bipolar mood disorders and those who used psychiatric medicines.

*I do not use modern medicine, because medical cannabis makes me feel better, healthier; so, I stopped modern medicine. Using too much modern medicine is not good for our liver and kidney. But it’s OK to use medical cannabis, even when we use too much there is less impact than from modern medicines. (NGO06)*

*Chemotherapy changes our tastebuds. Cannabis makes us feel sweater in the mouth and improves our tastebuds, so we can eat more. Cannabis should be taken along with chemo or radiotherapy. (FD02)*

*I advise DM patients to not use cannabis oil with medicine received from the hospital. If they faint, they should stop either modern medicine or cannabis, because their blood sugar may drop too much as cannabis washes out sugar in our body. (FD03)*

*Patients with irregular heartbeats cannot use cannabis. Bipolar patients and other psychiatric diseases should be careful too. If wanting to use medical cannabis it should be at a very low dosage, stop modern medicine or make a 2-3-hour interval between cannabis and modern medicine. (FD02)*

*Progression of illness and side effects.* After use of medical cannabis, most patients felt markedly better or cured, while some no longer returned to the hospital for treatment. The participants, either providers or users, believed that cannabis helped users to acquire deep sleep, an increase in appetite and a decreased pain, so the patients’ health and quality of life improved, their symptoms subsided, or their disease was cured. A number of folk doctors were aware of the negative health effects of cannabis; for example, intoxication when overdosing and toxicity when using low-quality products that were contaminated with insecticides or other toxic agents. However, most patients and folk doctors we interviewed had never experienced adverse effects of cannabis use by themselves.

*The negative impact of cannabis is zero. I never see anyone with shock, death or progressive diseases because of cannabis. (FD03)*

*Patients with skin diseases can use cannabis oil. Some who have whole body psoriasis get better after using cannabis oil, soap and cream for one month. Itching and lesions disappear. (FD03)*

*The obvious change I have seen is in cancer patients. Patients feel hopeful. People generally think that cancer patients must die, get chemo or radio. However, using cannabis, patients just stay happily at home and drop cannabis oil. This makes them feel more energetic. Cannabis activates the thought system in that they can survive. We advise them to use medical cannabis along with modern medicine. Cannabis is just an alternative. (NGO02)*

## Discussion

Our paper provides insights on the experiences of folk healers and illegal providers in providing medical cannabis treatment. It was found that unlicensed providers were more popular than licensed practitioners in government medical cannabis clinics. Warmth, friendliness, supportiveness, non-judgmental attitudes and all-time accessibility, with free or low-cost treatment, made those folk healers or not-for-profit providers in this study well accepted by their patients. This led them to continue their practice, despite the availability of medical cannabis clinics in MoPH hospitals all over the country. The providers in this study used cannabis products to treat all diseases, such as skin, eyes, HIV/AIDS, and non-communicable diseases as well as all kinds of cancers. Additionally, it was believed that it was effective, with no or minimal adverse effects. They chose the product forms and dosages based on the patient’s symptoms, and some even tailor-made the medicine concentration to suit the patients’ condition. These findings mirror what was found from our cross-sectional studies among medical cannabis users, in that they sought medical cannabis products and treatment from illegal or unlicensed sources more than from the governments’ official medical cannabis clinics, and used cannabis for treatment of all diseases or symptoms. ^7^

Studies in Canada and the USA found that healthcare professionals felt reluctant and ambivalent in using cannabis for medical purposes, because of a lack of knowledge and concern about its adverse effects. ^5^^,^^6^^,^^10^^,^^11^ However, our participants, both providers and patients, reported few or none of these concerns. The perception of medical cannabis as the last resort for those with terminal illness under limited access to modern healthcare systems determined a lack of concern. To our knowledge, there has not been a published study on attitudes and practices of medical doctors towards medical cannabis in Thailand. If it is similar to what was found in Canada and the USA, we could perceive that unlicensed or illegal providers could fill the gap of treatment for most patients of medical cannabis.

Similar to other studies, ^12^^–^^14^ medical cannabis use was common among cancer patients. Both providers and patients in this study believed cannabis was good for the treatment of cancers, by alleviating pain, anorexia, nausea and sleep difficulty as well as improving body systems to tolerate modern treatment side effects; with few or no adverse effects being reported. Providers’ trustworthiness, caring attitude and easy accessibility were the main ingredients for these patients to adhere to their treatment and a feeling of improvement from their suffering. Thus, it is expected that many cancer patients - especially those in the terminal stage in Thailand - will turn to unlicensed or illegal medical cannabis providers, and cannabis use will continue to expand nationally. It has been unfortunately observed that many clinicians in the public healthcare system have limited knowledge concerning medical cannabis, and this results in patients turning to unlicensed providers who are willing to provide treatment. As such, our findings underline the need for oncologists or palliative care clinicians to be prepared to discuss with their patients regarding medical cannabis, or to recommend it clinically. Evidence to inform cancer treatment guidelines on potential benefits and harms of medical cannabis, matched with a Thai context is also required.

Our study suggests that some patients will continue receiving medical cannabis treatment and products from unlicensed or illegal providers, despite licensed providers being available. This indicates the need to expand medical cannabis services in MoPH hospitals, and the requirement for reliable information for patients to access. The profusion of non-scientific information from websites, social media and community interaction reflects inadequate scientific information on efficacy and current healthcare service systems. However, increasing evidence of the benefits and safety of medical cannabis has appeared in international literature. ^15^^–^^18^ Capacity development and certification of unlicensed providers, and a simplified version of correct scientific evidence for patients to understand the risks and benefits of use are imperative. As reported by the participants of this study, some underground cannabis businesses who produce and sell expensive, but poor-quality or fake products also exist. There needs to be a system to monitor and to control quality, price and safety of medical cannabis products sold in the market place, which will be most beneficial to users who need it. Only through evidence-based interventions in healthcare systems and clear public health policies of medical cannabis, can success in medical cannabis service provisions be ensured with best outcomes of safety and efficacy.

### Limitations

Only data from a limited number of medical cannabis providers and users were included. Although we recruited sample to saturation and stopped interviewing new participants when no additional themes emerged in our last interview, our sample might subject to volunteer bias as most of the respondents were positive towards medical cannabis use. This may have led them to report only the positive side of cannabis use, and the unlicensed or illegal services. Snowball sampling was used to reach the participants, so this might limit the participants to the group of those who had similar values towards medical cannabis, and overrepresent supporters of medical cannabis.

## Conclusion

Unlicensed or illegal medical cannabis providers were still, and tended to remain popular, in Thailand. Patients regarded them as a last, dependable and trustful resource under limited access to public healthcare systems. Significantly more attention should be paid on increasing the capacity of medical cannabis service systems within public health hospitals. Additionally, certification of unlicensed providers, so as to integrate them into a regulated system where quality assurance can be maintained, is required. Furthermore, clear scientific information should be disseminated to patients who require the use of cannabis for treatment of their illnesses.

## Data availability

### Underlying data

The interview transcripts cannot be shared publicly as they contain personal and sensitive information, which could identify the participants. The interview transcripts are all in Thai. Anyone wishing to read the summary report of the data, including quotes may contact the corresponding author ( savitree.a@psu.ac.th), who will do translation of the parts requested.

### Extended data

Open Science Framework: Views and practices on medical cannabis of unlicensed providers in Thailand: a qualitative study. https://doi.org/10.17605/OSF.IO/PBRHJ. ^19^

This project contains the following extended data:

- Interview guide-providers.pdf
- Interview guide-users.pdf

Data are available under the terms of the Creative Commons Zero “No rights reserved” data waiver (CC0 1.0 Public domain dedication).
