# Supplementary material for: Views and practices on medical cannabis of unlicensed providers in Thailand: a qualitative study
Source: F1000Res. 2024 Aug 27;11:365. Originally published 2022 Mar 29. [Version 4] doi: 10.12688/f1000research.110367.4 (PMC11362744; doi:10.12688/f1000research.110367.4)
Supplement: Peer review history

# Reviewer report: Reviewer response for version 4

**DOI:** 10.5256/f1000research.170500.r317688

**Published:** 2024-08-29

**Authors:** Thomas Kerr

**Affiliations:** 1 The University of British Columbia, Vancouver, British Columbia, Canada

I have no further comments.

Is the work clearly and accurately presented and does it cite the current literature?

Yes

If applicable, is the statistical analysis and its interpretation appropriate?

Yes

Are all the source data underlying the results available to ensure full reproducibility?

Yes

Is the study design appropriate and is the work technically sound?

Yes

Are the conclusions drawn adequately supported by the results?

Yes

Are sufficient details of methods and analysis provided to allow replication by others?

Yes

Reviewer Expertise:

substance use epidemiology and public health

I confirm that I have read this submission and believe that I have an appropriate level of expertise to confirm that it is of an acceptable scientific standard.

# Reviewer report: Reviewer response for version 3

**DOI:** 10.5256/f1000research.159429.r303294

**Published:** 2024-08-07

**Authors:** Thomas Kerr

**Affiliations:** 1 The University of British Columbia, Vancouver, British Columbia, Canada

This is an interesting and well conducted study on an important topic. I congratulate the authors on a fine piece of work. The study nicely details reasons why medical cannabis users in Thailand continue to rely on unlicensed dealers, and how providers practice. One issue with the study is the inclusion of second objective which looks at how unlicensed providers practiced medical cannabis treatment. This makes the study quite long and its unclear how this relates to the first objective, which seems more interesting. Given the large size of the sample (large for a qualitative study), one wonders if that could be saved for a second paper. A small number of detailed comments are provided below:

  

- I suggest the authors use the term “theme” instead of “reasons” to describe their results, as is common practice in qualitative research

 

- The authors may wish to refer to “contemporary Western medicine” when contrasting it with “Thai traditional medicine” in the introduction.

 

- Can you specify how many follow-up interviews were done and with which participants?

 

- Can the author provide some justification for including those who are legal providers?

Why would they have insight into why some continue to rely on illegal sources?

  

- Can the authors provide some explanation for the low number of women in the study sample?

 

- The acronyms after each quote are difficult to discern. Can these be spelled out somewhere?

 

- It seems that the study covers a second topic – how unlicensed providers practiced medical cannabis treatment – but the themes arising from this are not listed in the abstract as with the first objective.  

 

- Previous literature on physician’s reluctance to prescribe cannabis also highlights concerns about a lack of information on dosing, impacts and routes of administration, and this should likely be added to the related section in the discussion.

 

- The section recommending that physicians be better prepared to talk about medicinal cannabis should also perhaps highlight that unlicensed providers should also be better educated about uses and contraindications of cannabis use given some of the inaccurate and potentially harmful claims evident in the qualitative quotes. Perhaps the authors could elaborate on this sentence to make this point: “ *Only through evidence-based interventions in healthcare systems and clear public health policies of medical cannabis, can success in medical cannabis service provisions be ensured with best outcomes of safety and efficacy.”*

 

- Could the authors add some commentary to the discussion about the current political/policy climate surrounding cannabis access in Thailand? I understand there is a move to reclassify cannabis to its previous status.

Is the work clearly and accurately presented and does it cite the current literature?

Yes

If applicable, is the statistical analysis and its interpretation appropriate?

Yes

Are all the source data underlying the results available to ensure full reproducibility?

Yes

Is the study design appropriate and is the work technically sound?

Yes

Are the conclusions drawn adequately supported by the results?

Yes

Are sufficient details of methods and analysis provided to allow replication by others?

Yes

Reviewer Expertise:

substance use epidemiology and public health

I confirm that I have read this submission and believe that I have an appropriate level of expertise to confirm that it is of an acceptable scientific standard, however I have significant reservations, as outlined above.

# Response

**Published:** 2024-08-16

**Authors:** Sawitri Assanangkornchai

**Affiliations:** Prince of Songkla University, Thailand

We sincerely appreciate your time in reviewing our manuscript and providing such thoughtful and constructive feedback. Your comments have been very helpful in improving the clarity and focus of our paper.

 We agree with your comment that the inclusion of the second objective (how unlicensed providers practised medical cannabis treatment) makes the paper quite long and somewhat disjointed from the main focus on why patients continue to rely on unlicensed dealers. Ideally, we would have addressed this issue by saving the results of the second objective for a separate paper, as you suggested. However, as the paper is already published, this is unfortunately not possible now. We hope you understand our position on this matter.

  

- I suggest the authors use the term “theme” instead of “reasons” to describe their results, as is common practice in qualitative research

 
**Response:** Thank you for your suggestion. We have changed it accordingly.

  

- The authors may wish to refer to “contemporary Western medicine” when contrasting it with “Thai traditional medicine” in the introduction.

  
**Response:** Thank you for your suggestion. We have changed it accordingly.

  

- Can you specify how many follow-up interviews were done and with which participants?

 
**Response: **Thank you for your feedback. We appreciate your bringing this to our attention.

 We understand that the number of follow-up interviews may not be immediately apparent from the data presented. We have clarified this point in the revised manuscript.

 In our data collection, we visited almost all participants more than once. This was because, on the first visit, we needed to establish rapport and build trust and might not get much data. The follow-up visits were then necessary to obtain accurate and in-depth information.

  

- Can the author provide some justification for including those who are legal providers?

Why would they have insight into why some continue to rely on illegal sources?

**Response:** We appreciate your question regarding including licensed providers in our study. We understand the concern about their potential lack of insight into why patients continue to rely on illegal sources. However, we believe that including licensed providers in our analysis was crucial for some reasons, such as helping us understand potential barriers that prevented patients from accessing legal and regulated care and identifying gaps in services -whether the practices of licensed providers were adequately meeting the needs of patients. We have included this justification in the revised manuscript.

  

- Can the authors provide some explanation for the low number of women in the study sample?

 
**Response:** We appreciate your query regarding the low number of women in our study sample. We acknowledge that women are less represented than men in both the unlicensed provider and user groups. However, in Thailand, as our data indicates, the majority of unlicensed providers were men. Societal roles and expectations might influence women's involvement in medical cannabis-related activities, both as providers and users. This gender imbalance among providers and users likely influenced the overall gender distribution of our sample.

 Furthermore, we employed a purposive sampling technique, relying on key informants to identify potential participants. Unfortunately, the majority of contacts provided by these informants were male providers, contributing to the overrepresentation of men in our sample. We have added this explanation to the “Limitations” section of the revised version.

  

- The acronyms after each quote are difficult to discern. Can these be spelled out somewhere?

   
**Response:** Thank you for your suggestion. We have changed all accordingly.

- It seems that the study covers a second topic – how unlicensed providers practiced medical cannabis treatment – but the themes arising from this are not listed in the abstract as with the first objective.  

 
**Response:** Thank you for your feedback. We have added it accordingly.

  

- Previous literature on physician’s reluctance to prescribe cannabis also highlights concerns about a lack of information on dosing, impacts and routes of administration, and this should likely be added to the related section in the discussion.

 
**Response: **Thank you for your suggestion. We have added it accordingly.

  

- The section recommending that physicians be better prepared to talk about medicinal cannabis should also perhaps highlight that unlicensed providers should also be better educated about uses and contraindications of cannabis use given some of the inaccurate and potentially harmful claims evident in the qualitative quotes. Perhaps the authors could elaborate on this sentence to make this point: “ *Only through evidence-based interventions in healthcare systems and clear public health policies of medical cannabis, can success in medical cannabis service provisions be ensured with best outcomes of safety and efficacy.”*

 
**Response:** Thank you for your feedback. We have revised the discussion accordingly.

  

- Could the authors add some commentary to the discussion about the current political/policy climate surrounding cannabis access in Thailand? I understand there is a move to reclassify cannabis to its previous status.

**Response:** Thank you for your feedback. We appreciate you bringing this to our attention. We have added a paragraph discussing this point to the revised manuscript.

 We are truly grateful for the reviewer’s positive assessment and valuable recommendations. Your feedback has significantly enhanced our work.

# Reviewer report: Reviewer response for version 3

**DOI:** 10.5256/f1000research.159429.r227708

**Published:** 2024-05-25

**Authors:** Davide Fortin

**Affiliations:** 1 Economic and Social Sciences, Health Systems and Medical Informatics (SESSTIM), Aix-Marseille University, Marseille, France; 2 Barcelona Institute for Global Health (ISGlobal), Hospital Clínc, University of Barcelona, Barcelona, Spain, Spain

This article can be approved.

Is the work clearly and accurately presented and does it cite the current literature?

Partly

If applicable, is the statistical analysis and its interpretation appropriate?

Not applicable

Are all the source data underlying the results available to ensure full reproducibility?

No source data required

Is the study design appropriate and is the work technically sound?

Yes

Are the conclusions drawn adequately supported by the results?

Partly

Are sufficient details of methods and analysis provided to allow replication by others?

Yes

Reviewer Expertise:

NA

I confirm that I have read this submission and believe that I have an appropriate level of expertise to confirm that it is of an acceptable scientific standard.

# Reviewer report: Reviewer response for version 2

**DOI:** 10.5256/f1000research.157733.r218877

**Published:** 2023-11-01

**Authors:** Davide Fortin

**Affiliations:** 1 Economic and Social Sciences, Health Systems and Medical Informatics (SESSTIM), Aix-Marseille University, Marseille, France; 2 Barcelona Institute for Global Health (ISGlobal), Hospital Clínc, University of Barcelona, Barcelona, Spain, Spain

This version is much better.

  I still cannot find any mention in the article about the following points:

1. Is it (or was it during the study period) possible to buy cannabis flowers as therapy, or is it only possible to buy cannabis extracts and other oral formulations?
2. About the technical and economic barriers which limits the scientific evidence related to cannabis (Fortin and Massin, 2019)

Is the work clearly and accurately presented and does it cite the current literature?

Partly

If applicable, is the statistical analysis and its interpretation appropriate?

Not applicable

Are all the source data underlying the results available to ensure full reproducibility?

No source data required

Is the study design appropriate and is the work technically sound?

Yes

Are the conclusions drawn adequately supported by the results?

Partly

Are sufficient details of methods and analysis provided to allow replication by others?

Yes

Reviewer Expertise:

NA

I confirm that I have read this submission and believe that I have an appropriate level of expertise to confirm that it is of an acceptable scientific standard, however I have significant reservations, as outlined above.

# Response

**Published:** 2023-11-27

**Authors:** Sawitri Assanangkornchai

**Affiliations:** Prince of Songkla University, Thailand

Thank you for your further comments.

 1. We have added some information on the availability of cannabis flowers during the study period as follows.

 - **Introduction, Paragraph 4:** "Our study, conducted during the first year after the legalization of medical cannabis use in Thailand, found ... Crude oil extract (unidentified tetrahydrocannabinol (THC) or cannabidiol (CBD) content) and raw plants ﻿(flowers, leaves or whole plants with roots and stems) were reported as ﻿the most common form of consumption by medical cannabis users."

 - **Results, under Subheading** ***Products and dosing:** "*The products forms were various, such as ..., tea made from dried raw plants, including flowers and whole plants, ...  Some folk healers also prescribed dried plants or flowers for smoking. These products were mostly obtained from illegal sources such as underground traders and home growers."

 2. We have added some discussion regarding the technical and economic barriers which limit the scientific evidence related to cannabis as follows.

 -Introduction, Paragraph 3: " Given the significant economic and technological obstacles to proving the effectiveness of medical cannabis [1], medical conditions with strong scientific evidence for the efficacy of cannabis that is based on randomized clinical trials on which physicians use to base their decisions are limited. ^9 ,^ ^10^ Fortin and Massin provided reasons for these barriers on the following four points: 1) The entourage effect would make the herbal form much more effective than single cannabinoids; 2) It seems difficult to prove the effectiveness of herbal cannabis through randomized controlled trials; 3) Even if it were possible, there would not be any financial incentive to carry out such research because it could not be patentable; 4) It seems unlikely that public solutions will address this issue quickly and affordably [1]."

# Reviewer report: Reviewer response for version 1

**DOI:** 10.5256/f1000research.121965.r202145

**Published:** 2023-10-03

**Authors:** Davide Fortin

**Affiliations:** 1 Economic and Social Sciences, Health Systems and Medical Informatics (SESSTIM), Aix-Marseille University, Marseille, France; 2 Barcelona Institute for Global Health (ISGlobal), Hospital Clínc, University of Barcelona, Barcelona, Spain, Spain

REPORT " *Views and practices on medical cannabis of unlicensed* *providers in Thailand: a qualitative study"*

 In a market which legalize the distribution of cannabis for medical purposes, the reason why patients still choose to buy cannabis illicitly is an interesting topic of research. In the article, the authors study this phenomenon during the first year after the legalization of ‘medical cannabis’ in Thailand. To do so, the authors perform interviews with medical cannabis providers and users. They identified six reasons behind the popularity of unlicensed provider, namely accessibility, familiarity with the providers prior to legalization, favourable character of the providers, affordability, quality and lack of knowledge towards cannabis from healthcare professionals.

 The paper is written quite decently, and the way the healthcare system in Thailand has integrated medical cannabis through three different groups of products is of interest for other countries with similar approaches to traditional medicine.

 A major issue is that it does not highlight enough the fact that interviews were taken during the first year after the passage of the law, when not only healthcare system is unprepared to prescribe to patients, but also few products are available and those which are distributed are thus likely to be quite expensive. This affect both patients from using them and physicians to prescribe it when they are aware of cheaper options to treat the medical condition. To help readers to better understand the research context, this fact should be probably not only be included in the abstract, but also the reason of low affordability and accessibility should be the first mentioned behind the choice of patients to go to unlicensed providers.

 I have a few other concerns which I believe need some attention and would help improve the paper :

- In the second paragraph, it is mentioned that “access to registered products for patients had been difficult as indications for prescription were limited and regulations regarding possession as well as production of medical cannabis were constricted.”. I think it should expanded to understand whether there is a limited number of medical conditions which allow the prescription of medical cannabis based on the existence of randomized clinical trials proving its efficacy. From the results, I understand insomnia is not consider a condition for the prescription, but the rationale behind which condition is accepted should be clearly identified in the text.
- It is not clear whether one of the three types of medical cannabis products which can be prescribe include flowers (or inflorescence). If this is not the case, it should be clearly stated as this type of products is the most used among countries which legalize the medical use of cannabis (e.g. Canada, US). In parallel, it should be also clarified whether some of the unlicensed producers were selling flowers and/or resins, or if they are only selling non-smokable products (If they sell flowers, this should be another reason why they are popular, the fact that they have a different type of medical cannabis product).
-  In the third paragraph, the authors mention “Furthermore, studies in Canada and the United States of America (USA), where medical cannabis is legally available, indicated that physicians felt reluctance or ambivalent to authorize cannabis use for their patients, because of either a lack of knowledge or unfamiliarity with pharmacology, formulations, dosing of cannabis, lack of product standardization, lack of research examining the effectiveness and risks of cannabis use, and uncertainty regarding the policies.”. Considering the increased in research in recent years related to cannabis (Ng and Chang, 2022), the real issue relates to the lack of Randomized Clinical Trials (as this is normally what physicians use to base their decision) which are hampered from technical and economic barriers related to medical cannabis (Fortin and Massin, 2020). This should be clearly explained in the text.
- About the lack of knowledge from physicians, Hagani et al. (2021) should be included as it studied the gaps in perceptions toward MC between patients and health professionals.
- In the literature, Fortin (2022) should be added as it models the competition between healthcare system and illicit market for medical cannabis and provide some additional solutions to reduce the number of patients who buy their treatment in the illicit market.
- When talking about the issue of low availability, Wadsworth et al. (2021) should be added as they show that retail availability was associated with last purchasing dried flower legally among past-year dried flower purchasers.

Other points to be considered below:

- In the second paragraph, they mention the first type of product saying “registered drugs per the new Narcotics Act”. I don’t think that the wording “per the” is grammatically correct.
- Instead of using the term “users”, the author should use the term “patients” given that we assume they are using cannabis for medical purposes.
- I think this sentence should be rephrased to be more easily understood :“The perception of medical cannabis as the last resort for those with terminal illness under limited access to modern healthcare systems determined a lack of concern.”

Is the work clearly and accurately presented and does it cite the current literature?

Partly

If applicable, is the statistical analysis and its interpretation appropriate?

Not applicable

Are all the source data underlying the results available to ensure full reproducibility?

No source data required

Is the study design appropriate and is the work technically sound?

Yes

Are the conclusions drawn adequately supported by the results?

Partly

Are sufficient details of methods and analysis provided to allow replication by others?

Yes

Reviewer Expertise:

Cannabis Policy and Economics, Health Economics, Industrial Organization, Cost-Effectiveness Analysis, Patient-Reported Outcomes

I confirm that I have read this submission and believe that I have an appropriate level of expertise to confirm that it is of an acceptable scientific standard, however I have significant reservations, as outlined above.

## References

[1] : Retail availability and legal purchases of dried flower in Canada post-legalization. *Drug Alcohol Depend* .2021;225: 10.1016/j.drugalcdep.2021.108794 108794 10.1016/j.drugalcdep.2021.108794 34098382

[2] : Attitudes Toward the Use of Medical Cannabis and the Perceived Efficacy, Side-effects and Risks: A Survey of Patients, Nurses and Physicians. *J Psychoactive Drugs* .2022;54(5) : 10.1080/02791072.2021.2009598 393-402 10.1080/02791072.2021.2009598 34893011

[3] : A bibliometric analysis of the cannabis and cannabinoid research literature. *J Cannabis Res* .2022;4(1) : 10.1186/s42238-022-00133-0 25 10.1186/s42238-022-00133-0 35610633 PMC9131698

[4] : CSC + 2.2022; 10.4324/9781003055679-8 133-154 10.4324/9781003055679-8

[5] : Medical cannabis: thinking out of the box of the healthcare system. *Journal de gestion et d'economie de la sante* .2020;2(2) :110-118

# Response

**Published:** 2023-10-12

**Authors:** Sawitri Assanangkornchai

**Affiliations:** Prince of Songkla University, Thailand

Thank you for your valuable comments and suggestions.

 As suggested, we will add more information in the introduction part of the manuscript on the timing of the study and how it affected the health system, the availability of cannabis-based products, and physician prescription practice. A list of medical conditions approved by the Thai Ministry of Public Health for cannabis treatment and those with supported evidence of efficacy from randomized clinical trials will be added. We will also explain if cannabis flowers and resins were included in the prescribed and unlicensed medical cannabis products in the introduction and discussion parts. Furthermore, the references suggested by the reviewer will be cited to support the explanation and discussion of the related issues. Lastly, we will check the grammatical correctness and rephrase the unclear sentence.
